# Transcriptional and Behavioral Responses of Zebrafish Larvae to Microcystin-LR Exposure

**DOI:** 10.3390/ijms18020365

**Published:** 2017-02-09

**Authors:** Eleni Tzima, Iliana Serifi, Ioanna Tsikari, Ainhoa Alzualde, Ioannis Leonardos, Thomais Papamarcaki

**Affiliations:** 1 Laboratory of Biological Chemistry, Medical School, University of Ioannina, 45110 Ioannina, Greece; elenatzima@gmail.com (E.T.); iliana.serifi@gmail.com (I.S.); juanita_tsiki@hotmail.com (I.T.); 2 Division of Biomedical Research, Foundation for Research and Technology-Hellas, Institute of Molecular Biology and Biotechnology, 45110 Ιοannina, Greece; 3 BIOBIDE, Paseo Mikeletegi 56, 20009 San Sebastian, Spain; alzualde@biobide.es; 4 Laboratory of Zoology, Department of Biological Applications and Technologies, University of Ioannina, 45110 Ioannina, Greece; ileonard@uoi.gr

**Keywords:** MCLR, zebrafish, gene expression, behavior, visual cycle

## Abstract

Microcystins are cyclic heptapeptides that constitute a diverse group of toxins produced by cyanobacteria. One of the most toxic variants of this family is microcystin-LR (MCLR) which is a potent inhibitor of protein phosphatase 2A (PP2A) and induces cytoskeleton alterations. In this study, zebrafish larvae exposed to 500 μg/L of MCLR for four days exhibited a 40% reduction of PP2A activity compared to the controls, indicating early effects of the toxin. Gene expression profiling of the MCLR-exposed larvae using microarray analysis revealed that keratin 96 (*krt96*) was the most downregulated gene, consistent with the well-documented effects of MCLR on cytoskeleton structure. In addition, our analysis revealed upregulation in all genes encoding for the enzymes of the retinal visual cycle, including *rpe65a* (retinal pigment epithelium-specific protein 65a), which is critical for the larval vision. Quantitative real-time PCR (qPCR) analysis confirmed the microarray data, showing that *rpe65a* was significantly upregulated at 50 μg/L and 500 μg/L MCLR in a dose-dependent manner. Consistent with the microarray data, MCLR-treated larvae displayed behavioral alterations such as weakening response to the sudden darkness and hypoactivity in the dark. Our work reveals new molecular targets for MCLR and provides further insights into the molecular mechanisms of MCLR toxicity during early development.

## 1. Introduction

Cyanotoxins are secondary metabolites produced by several species of cyanobacteria that exist in freshwater lakes and coastal marine ecosystems due to eutrophication. They are classified into three broad groups according to their chemical structure as cyclic peptides, alkaloids, and lipopolysaccharides [1]. Microcystins are monocyclic heptapeptides produced by several members of cyanobacterial genera including *Microcystis*, *Anabaena*, and *Planktothrix* [2]. To date, more than 90 variants have been identified, and among them microcystin-LR (MCLR) is the most common and toxic member [2,3]. Microcystin-LR is extremely stable under physiological conditions and resistant to chemical hydrolysis or oxidation. A slow degradation occurs either at high temperatures (40 °C) or high/low pH [4]. The toxin can also be degraded by bacterial metalloproteases, called microcystinases, but the bacterial species that produce these enzymes are often absent from surface water [5].

In aquatic environments the concentrations of MCLR vary from traces to 1800 μg/L or even higher during periods of cyanobacterial blooms [6]. Upon cyanobacteria cell death or lysis, MCLR is released into the aquatic environment and the exposure of fish may occur either by oral uptake of toxin-containing cells or via epithelium surfaces that are immersed in the polluted water [7]. Organic anion transporters (OATPs) are specifically required for active uptake of MCLR into hepatocytes and epithelial cells [8]. These transporters are highly expressed in the liver and are also found in the heart, lung, spleen, pancreas, and brain [9].

Regarding fish, MCLR toxicity develops at concentrations in the range of μg/L, depending on the aquatic species, the developmental stage, and the exposure route (intraperitoneal injection, feeding, or immersion) [7,10,11]. During early development, the toxin can disrupt embryonic hatching and growth rate, causing morphological abnormalities such as small head and curved body. It also affects the heart rate and the physiological hepatocyte structure [12]. When fish are exposed to high concentrations of MCLR (>1000 μg/L), hepatocytes die through necrosis, which often leads to tissue disruption and liver failure [12]. 

Previous studies have proposed that MCLR hepatotoxicity could be due to hyperphosphorylation of actin-associated proteins, which leads to cytoskeleton breakdown and ultimately disrupts the cellular architecture inducing apoptosis in liver [13,14]. Recent evidence suggests that all changes in the phosphorylation status of the cytoskeleton proteins may be attributed to the inhibition of the serine/threonine protein phosphatase 2A (PP2A) by MCLR [15,16]. Protein phosphatase 2A plays essential roles in the control of many biological processes, including cell growth, proliferation, apoptosis, and differentiation, and its activity should be very tightly regulated to maintain the cellular homeostasis [17,18]. Crystallographic experiments revealed that MCLR binds specifically to the active site of the catalytic subunit of PP2A, inhibiting its enzymatic activity with *Ki* value in the picomolar range [19]. Subsequent studies have shown that alterations of PP2A activity by MCLR is an early event during the toxin exposure and can be considered as one of the main mechanisms of MCLR toxicity [20]. 

Fish exposed to microcystins have developed detoxification mechanisms to resist the toxin risks. The glutathione pathway is an important biochemical mechanism for the formation of glutathione (GSH) conjugates that increases the water-solubility of MCLR, mediating both its metabolism and elimination [21,22]. Besides the enzymatic activity of PP2A and glutathione transferase, MCLR also affects acetylocholinesterase (AChE), an enzyme that plays key roles in neurotransmitter systems and is widely used as a bioindicator of environmental toxicity [23]. Recently, Zeng et al. have shown that MCLR exposure is accompanied by reactive oxygen species (ROS) production that consequently triggers apoptosis in developing zebrafish embryos in a caspase-dependent manner [24]. 

Zebrafish has emerged as a prominent organism for investigating vertebrate development and is widely used for environmental and toxicological studies [25,26]. The nervous system of larval fish is suitable for studies of fundamental neuronal pathways because zebrafish have broad homologies to mice and humans with respect to brain patterning, and the structure/function of several neural systems [27]. Recently, a number of neurobehavioral locomotor assays, including learning and memory, have been developed in zebrafish to complement genetic studies [28,29,30,31]. In the present study, we investigated the transcriptional and behavioral responses of zebrafish larvae to MCLR exposure and searched for biochemical pathways affected by the toxin at sublethal concentrations of MCLR. 

## 2. Results

### 2.1. Alterations of Protein Phosphatase 2A Activity in Zebrafish Larvae Following Exposure to Microcystin-LR

**Figure 1:**
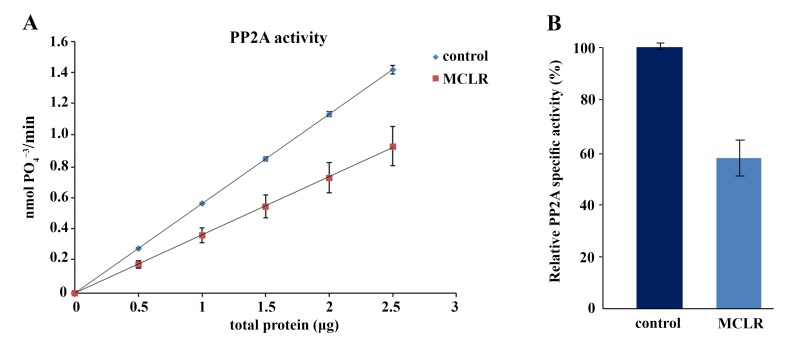
Effects of microcystin-LR (MCLR) exposure on protein phosphatase 2A (PP2A) activity. (**A**) PP2A activity was measured in control and larvae exposed to 500 μg/L of MCLR, as described in the Materials and Methods section. Protein phosphatase 2A activity was expressed as nmol of free PO_4_^−3^ released per min using increasing amounts of total protein (μg). The data are indicated as mean ± SE obtained from three independent experiments (*p* < 0.05); (**B**) Relative PP2A specific activity of MCLR group vs. control. Control was set to 100%.

To understand the effects of MCLR on early zebrafish development, three days post-fertilization (dpf) zebrafish larvae were exposed to different concentrations of purified MCLR, ranging from 50–500 μg/L for a period of four days. Morphological observations of the treated larvae showed that the highest concentration of 500 μg/L of MCLR did not cause death or any obvious morphological defects in the exposed animals (not shown). Under these experimental conditions, PP2A activity was markedly reduced in the treated larvae, consistent with the established inhibitory role of MCLR to PP2A [16,19] (Figure 1A). The reduction of PP2A activity in the MCLR-exposed larvae was calculated to be 42.4% ± 6.9% (Figure 1B). These results suggest that exposure of three dpf larvae to 500 μg/L of MCLR within four days can be used to assess early changes of gene expression in the treated fish. Thus, it was tempting to speculate if this dose, effective for a metabolic change without overt toxic consequences, can be used to detect early changes of gene expression in treated larvae.

### 2.2. Gene Expression Analysis 

To determine the global gene expression profile of larvae treated with 500 μg/L MCLR, we used DNA microarray analysis. Using a threshold of fold change ≥1.5 and *p* ≤ 0.05, the total number of the differentially expressed transcripts with known gene ID was 53, and among them, four genes were represented by two different transcripts: *tyr* (tyrosinase), *cp* (ceruloplasmin), *ttna* (titin a), *lgals1l1* (lectin, galactoside-binding, soluble, 1) (Table 1). All information concerning the differentially regulated genes and the function of the encoded proteins was obtained from the National Center for Biotechnology Information (NCBI). Our detailed analysis showed that *krt96* (keratin 96) and the cytoskeleton-associated *ttna* and *ttnb* (titin b) were among the most downregulated transcripts (Table 1), consistent with the well-documented effect of MCLR on cytoskeleton structure and dynamics [13,14,15,32,33]. In addition, most genes of the melanin synthesis pathway such as *tyr*, *dct* (dopachrome tautomerase), *tyrp1a* (tyrosinase-related protein 1a), *tyrp1b* (tyrosinase-related protein 1b) *pmela* (premelanosome protein a) and *pmelb* (premelanosome protein b) were upregulated by 1.55-, 2.13-, 1.51-, 1.70-, 1.72-, and 2.06-fold, respectively (Table 1). These microarray data have been confirmed by quantitative real-time PCR (qPCR) analysis revealing 4.30-fold upregulation of *tyr*, 5.84-fold of *dct*, 4.71-fold of *pmela*, and 11.36-fold of *pmelb* at 500 μg/L of MCLR exposure (*p* ≤ 0.05, data not shown).

**Figure 2:**
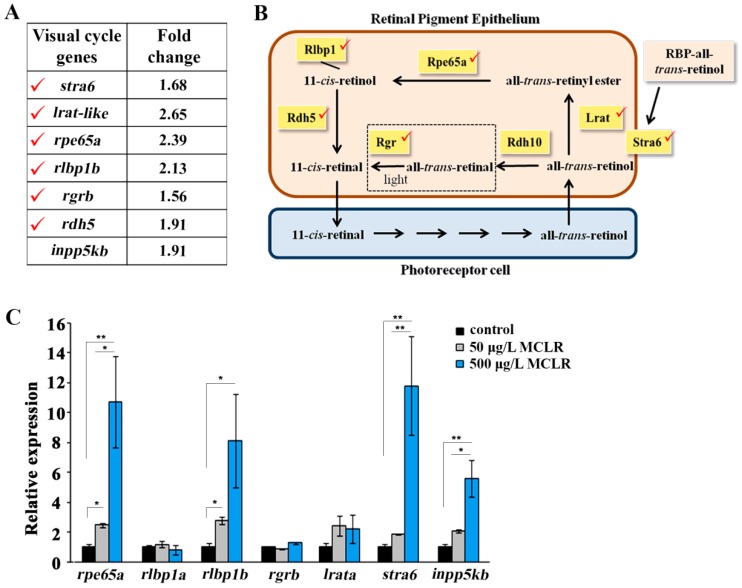
Gene expression profiling of MCLR exposed larvae (**A**) microarray analysis. Differentially expressed genes in zebrafish larvae treated with 500 μg/L of MCLR involved in the retinoid visual cycle, *p* < 0.05. (**B**) The retinoid visual cycle. Briefly, absorption of light by visual pigments causes isomerization of 11-*cis*-retinal to all-*trans*-retinal, resulting in phototransduction. All-*trans*-retinal is reduced to all-*trans*-retinol and transported into the retinal pigment epithelium (RPE). LRAT esterifies all-*trans*-retinol to all-*trans*-retinyl esters, which are further processed to 11-*cis*-retinol by RPE65. RLBP1 removes 11-*cis*-retinol from the reaction site to speed the isomerization. RDH5 converts 11-*cis*-retinol to 11-*cis*-retinal. STRA6 receptor mediates all-*trans*-retinol uptake through the plasma membrane. Red check marks indicate the visual cycle-related genes detected in MCLR-treated larvae by microarray analysis. (**C**) Validation of microarray data by quantitative real-time PCR (qPCR) analysis. Relative mRNA expression of rpe65a, rlbp1a, rlbp1b, rgrb, lrata, stra6, and inpp5kb (inositol polyphosphate-5-phosphatase Kb) at a four-day exposure of larvae to 50 μg/L and 500 μg/L of MCLR. Three biological replicates were run in triplicate. The mRNA expression levels are expressed in relation to the control which is set to 1. * *p* < 0.05 and ** *p* < 0.01. RBP: Retinol binding protein.

Our data also demonstrated that all the enzymes of the retinoid visual cycle were differentially expressed upon the exposure of three dpf larvae to 500 μg/L of MCLR (Figure 2A). The visual cycle takes place within the outer segments of the photoreceptors and the retinal pigment epithelium (RPE) and is constituted by a series of reactions that regenerate 11-*cis*-retinal from all-*trans*-retinol (outlined in Figure 2B). The critical and apparently rate-limiting step in the retinoid visual cycle is the conversion of the all-*trans* isomer to its 11-*cis* form by the retinal pigment epithelium-specific protein 65 kDa (RPE65) [34,35,36]. 

As indicated by the microarray results, the transcription levels of *rpe65a*, *rlbp1b*, *rgrb*, *rdh5*, *lrat-like*, and *stra6* were upregulated (Figure 2A). Moreover, *inpp5kb*—which encodes an enzyme with roles in the phosphoinositide metabolism, that regulates retina function [37]—showed upregulation by 1.91-fold (Table 1, Figure 2A). 

To check for early effects of MCLR on gene expression, we analyzed the levels of all the above genes at 50 μg/L and 500 μg/L of MCLR using qPCR analysis, which is a more sensitive and accurate method than microarrays (Figure 2C). In more detail, *rpe65a* was up-regulated by 2.48- and 10.80-fold, *rlbp1a* (NM_200705.1) 1.18-fold and 0.82-fold, *rlbp1b* (NM_205690.2) 2.76-fold and 8.09-fold, *lrata* (NM_001204131) 2.44- and 2.22-fold, *stra6* 1.86- and 11.85-fold and *inpp5kb* by 2.07-fold and 5.57-fold in larvae treated with 50 μg/L and 500 μg/L MCLR, respectively. 

Therefore, our results show that exposure of zebrafish larvae to MCLR affects the transcription levels of cytoskeleton-associated, melanin synthesis, and visual cycle genes that may in turn affect the physiological responses of the fish. 

### 2.3. Behavioral Alterations upon Microcystin-LR Exposure of Zebrafish Larvae

**Figure 3:**
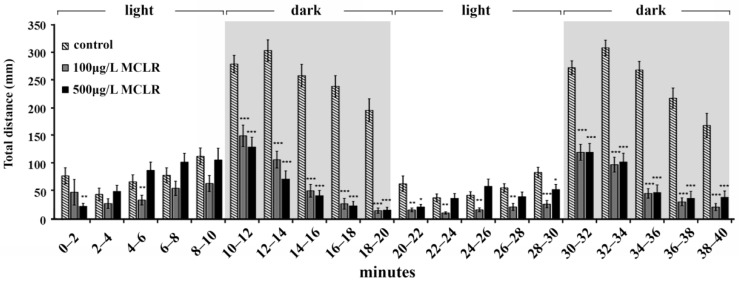
Behavioral patterns of MCLR-treated zebrafish larvae. The locomotor activity displayed by controls (0.05% ethanol) and treated larvae with 100 µg/L and 500 µg/L of MCLR is represented by the total distance covered in 2 min time bins. Tracking was performed under variable lighting conditions. Light phases correspond to 0–10 min and 20–30 min and dark phases to 10–20 min and 30–40 min time periods. * *p* < 0.05, ** *p* < 0.01, and *** *p* < 0.001 comparing each treatment to the control group.

Several studies have shown that MCLR accumulates in the brain [9] and exerts toxicity in the nervous system and impacts functions, including behavioral changes [23,38,39]. Furthermore, our data have shown that MCLR affects the transcriptional levels of all visual cycle genes. Therefore, it was tempting to investigate whether the MCLR exposed fish exhibited altered behavioral responsiveness to dark/light changes [40]. In the light, zebrafish normally have less locomotor activity than in the dark. After a sudden decrease in brightness, larvae respond with a rapid increase in movement [41] and exhibit a startle response when the light is turned back on again, followed by minimal movement in the light [40]. This reaction pattern is related to stress produced by the sudden darkness. To evaluate behavioral alterations in response to MCLR, three dpf larvae were exposed to 100 μg/L and 500 μg/L of MCLR for four days. After applying 10 min dark and 10 min light conditions in order to let larvae habituate to the system, tracking was performed at two rounds of 10 min light/10 min dark phases for 40 min. As shown in Figure 3, the behavior of larvae treated with the toxin was significantly different compared to the control group. As expected, control larvae reacted strongly against light/dark changes. On the contrary, larvae exposed to MCLR displayed a weakening reaction towards light changes, showing a constant hypoactivity during dark phases. This mitigated responsiveness of zebrafish larvae to environmental changes could be related to reduced stress and possibly to motor neuron and/or skeletal muscle functional defects caused by MCLR.

## 3. Discussion

In the present study, we have investigated the early effects of MCLR on zebrafish larvae at the molecular, transcriptional, and behavioral level. The responses of the larvae to sublethal concentrations of the toxin were estimated by measuring the reduction of PP2A activity, in accordance with the established inhibitory role of MCLR to PP2A [16,20]. The inhibition of PP2A activity may be used as a molecular indicator of the effects of MCLR. This suggestion is based on the fact that, at the molecular level, the determination of PP2A enzymatic activity evaluates more accurately the effects of MCLR that depend on the synergies of different factors; toxin bioaccumulation, exposure period, developmental stage of the organisms, and environmental conditions. Moreover, due to contaminant interference and large variations in recoveries of MCLR from tissue samples, the quantitation of MCLR in tissues by enzyme-linked immunosorbent assay (ELISA)-based strategies is more complicated and less reliable than in water samples (detection limit 0.1 μg/L) [22]. 

Our gene expression data of larvae exposed to MCLR identified upregulation of genes involved in the melanin synthesis pathway. This effect could be a compensatory mechanism for the treated individuals to circumvent the toxin threat and maintain their physiological status [42]. In addition, MCLR affected genes encoding for the enzymes of the retinoid visual cycle, suggesting molecular targets of MCLR in the visual pathway. Interestingly, most of the genes affected by MCLR are expressed in the RPE. For example, *rlbp1b*, which is localized in RPE, was upregulated in MCLR-treated larvae, while, *rlbp1a*, which is expressed only in Müller cells [43], was not among the differentially expressed transcripts in both microarray and qPCR analysis. The preference of MCLR action on RPE is interesting, because the pigment epithelium plays a critical role in the development of the retina and in some species it participates in the regeneration of the retinal tissue [44]. 

The mRNA changes of the above genes may imply an increase in enzyme production and/or activity. However, it is possible that some enzymes might indeed buffer fluctuations, whereas others might accelerate responses to MCLR. These parameters have not been analyzed in our work due to the lack of commercially available antibodies against the zebrafish proteins and the difficulties to assay for the interconnected isomerization reactions of the visual cycle. Since *rpe65* has been shown to be critical for the retinoid visual cycle and normal vision [34,35] and it responds transcriptionally to MCLR (our data), *rpe65a* transcript levels may predict a toxic outcome and can be used as an early biomarker of MCLR exposure. 

A previous microarray analysis by Rogers et al. in which three dpf larvae were exposed to 100, 1000 μg/L of MCLR, and *Microcystis aeruginosa* extract containing 4.5 μg/L of MCLR for four days, reported MCLR changes on genes related to cell signaling and development, cytoskeleton, immune function, and oxidative stress [45]. A comparison of our microarray results with the above study revealed one gene in common; *sepp1b* (selenoprotein P, plasma, 1b), which was reported to be downregulated in accordance with our data (Table 1). We have validated *sepp1b* expression in the 500 μg/L MCLR group by qPCR and found a 0.64-fold change. The observed variations between the two studies could be attributed to differences in MCLR concentrations. 

Our findings are novel and raise interesting questions concerning the mechanism of MCLR action. Protein phosphatase 2A activity, which is inhibited by MCLR, is a regulator of signal transduction pathways that coordinate the visual cycle [46]. Therefore, it is reasonable to hypothesize that upon entering the RPE cells, MCLR, through inhibition of PP2A, might affect cell signaling that orchestrates the recycling of 11-*cis*-retinal. In agreement with this hypothesis, a recent proteomic study by Li et al. has shown that MCLR exposure of zebrafish alters the expression of β-crystallins which are present in the lens and are involved in signal transduction [47]. 

Accumulated evidence demonstrates that MCLR can induce neurotoxicity in nematode, fish, birds, and mammals, and may result in behavioral changes [38,39]. However, the effects of MCLR on the behavior of fish are still largely unknown. A previous study by Baganz et al. recorded altered swimming capacity of adult zebrafish upon long-term treatment with MCLR [48]. Similar results have been obtained from adult zebrafish that were exposed to a cell culture of the microcystin-producing cyanobacterium *M. aeruginosa* in comparable experimental settings [49]. In our study, we have used the visual locomotor assay that tracks the locomotor responses to light onset and offset and relies on the visual pathway integrity, the nervous system development, and locomotor system development [50]. This behavioral analysis showed that MCLR-treated larvae displayed a weakening response upon rapid loss of light and reduced activity in the dark. It is known that sudden darkness produces a direct stress response to the zebrafish larvae that leads to an increased locomotor activity [40]. Based on our findings, one can speculate that the reduction of the physical activity of larvae exposed to MCLR after the sudden darkness could be due to reduced stress. Additionally, MCLR-treated larvae may display motor neuron and/or skeletal muscle functional defects. In support of this hypothesis, our microarray analysis showed that the muscle-associated *ttna* and *ttnb* were among the most downregulated transcripts (0.63-fold each). Furthermore, our experiments revealed that tubulin α-1b (tuba1b) transcript levels were significantly increased to 3.09-fold in the 50 μg/L MCLR group and to 7.78-fold in the 500 μg/L MCLR group (data not shown). Expression of tubulinα-1b is induced in the developing and regenerating central nervous system (CNS) of vertebrates and is considered to be a regulator of motor neuron synapse and axon function [51]. Future experiments using transgenic lines showing motoneuronal and/or muscular cell types could reveal in a more effective way possible effects of MCLR in these anatomical compartments. 

It has been established that behavioral profiling can characterize large classes of compounds and reveal information concerning their biological targets [52]. Moreover, the whole-animal behavioral responses can be measured effectively and characterize the sublethal exposure of fish to different molecules that is not accompanied by any obvious pathological effects [53]. Recently, the visual motor response (VMR) has been used for early safety assessment of oculotoxic drugs [54]. Therefore, our behavioral assay may be used to predict MCLR effects and to assess the toxicity risks.

In summary, in the present work we have (i) quantified the effect of MCLR on PP2A activity; (ii) identified the gene expression signature of the treated individuals using DNA microarrays; (iii) validated by qPCR analysis the gene expression changes at concentrations below the toxicity threshold; and (iv) complemented our toxicogenomic analysis with a high-throughput behavioral assay. Our findings provide new insights into the molecular mechanisms of microcystin-LR toxicity during early development.

## 4. Materials and Methods

### 4.1. Zebrafish Maintenance

Zebrafish maintenance, spawning, and embryo collection were performed under standard conditions. Briefly, zebrafish were cultivated in a closed flow-through system with charcoal-filtered tap water at 28 ± 0.5 °C in a 14:10 h light:dark photoperiod and were fed with commercial dry food distributed manually twice a day. Zebrafish were staged according to Kimmel et al. [55] and maintained in accordance with the European Directive 2010/63 for the protection of animals used for scientific purposes and the Recommended Guidelines for Zebrafish Husbandry Conditions. Animals were anaesthetized in 0.016% Tricaine MS-222, before sacrifice. The experimental protocols described in this study were carried out with zebrafish larvae up to seven dpf and were approved by the Foundation for Research and Technology-Hellas (FORTH) Ethics Committee (FEC) (Project Code 80821/2012).

### 4.2. Exposure of Zebrafish Larvae to Microcystin-LR

For exposure of zebrafish larvae to MCLR, 1 mg of pure MCLR (Enzo Life Sciences (New York, NY, USA) was dissolved in 100% ethanol and the MCLR/ethanol solution was added to fish water in different concentrations (50 μg/L and 500 μg/L). The final concentration of ethanol in fish water was ≤0.05% to avoid toxic effects. A control group was exposed to 0.05% ethanol. At three dpf, 55 larvae were exposed to 50 μg/L and 500 μg/L MCLR for four days in 250-mL glass beakers containing 100 mL of MCLR solution. These developmental stages were selected because at three dpf both the hatching period and the morphogenesis of the larvae are complete and by seven dpf, the free swimming larvae have a well-developed nervous system and the ability to perceive cues from the aquatic environment. Each concentration was studied using three independent biological replicates. 

### 4.3. Protein Phosphatase 2A Activity Assay

Seven dpf control and treated larvae (40 larvae per tube) were placed on ice and washed once with 50 mM Tris-HCl buffer (pH 7.5) and once with lysis buffer containing 50 mM Tris-HCl, 0.05% Triton X-100, 10% (*v*/*v*) glycerol, 0.5 mM phenylmethylsulfonyl fluoride (PMSF), 0.1 mM ethylenediaminetetraacetic acid (EDTA), and 0.02% (*v*/*v*) β-mercaptethanol. Then, larvae were incubated in lysis buffer supplemented with protease inhibitors cocktail (Roche) and 1 mM Na_2_VO_4_ (phosphatase inhibitor) for 40 min and homogenized using a 25-gauge syringe. Homogenates were centrifuged at 10,000× *g* for 3 min at 4 °C and the supernatants were collected. Whole-larvae lysates were passed through Sephadex G-50 columns (Pharmacia Biotech, Piscataway, NJ, USA) to remove free phosphate. The total protein concentration (before and after the column step) was determined by the Bradford method, using bovine serum albumin (BSA) as standard. 

To measure PP2A activity, different amounts of larvae lysates were incubated with the synthetic phospho-peptide substrate, RRApTVA (Enzo Life Sciences), which is specific for PP2A, for 30 min at 37 °C. The amount of released free phosphate was determined by measuring the absorbance of the molybdate-malachite green-phosphate complex (Sigma, St. Louis, MO, USA) at 630 nm. PP2A specific activity was expressed as nanomoles of free PO_4_^−3^ released per min per μg of total protein. Three independent experiments were performed.

### 4.4. RNA Preparation and cDNA Synthesis

Total RNA was extracted from control and larvae treated with 50 μg/L and 500 μg/L of MCLR (three biological replicates) using the RNeasy Mini Kit with DNase (Qiagen, Hilden, Germany) treatment to remove genomic DNA contamination. Quantification and quality analysis of RNA was performed via a Nanodrop spectrophotometer (Thermo Scientific Waltham, MA, USA) and confirmed by the Agilent 2100 Bioanalyzer (Agilent Technologies, Santa Clara, CA, USA). cDNA was synthesized using the SuperScript III First-Strand Synthesis SuperMix for qPCR (Invitrogen, Carlsbad, CA, USA) according to the manufacturer’s instructions.

### 4.5. Microarray Analysis

Affymetrix platform transcriptional microarray analysis was performed on RNA samples obtained from control and larvae treated with 500 μg/L of MCLR (three biological replicates from each group) using the Affymetrix GeneChip Zebrafish Gene 1.0 Arrays (Affymetrix, Santa Clara, CA, USA). RNA samples were processed and labeled for array hybridization using the Ambion WT Expression kit (Life Technologies, Carlsbad, CA, USA). Labeled, fragmented cDNA (Affymetrix GeneChip WT Terminal Labeling and Controls Kit) was hybridized to Zebrafish Gene 1.0 arrays (Affymetrix GeneChip Hybridization, Wash, and Stain Kit) for 16 h at 45 °C at 60 rpm using a GeneChip Hybridization Oven 640 (Affymetrix). Arrays were washed and stained using the Affymetrix Fluidics Station 450, and scanned using the Hewlett-Packard GeneArray Scanner 3000 7G (Palo Alto, CA, USA). Quality of array data was assessed using Expression Console (v 1.3) software (Affymetrix) prior to importing and analyzing the data in GeneSpring 13.0 (Agilent Technologies). Data was analyzed with settings for Affymetrix Expression experiment type, Exon analysis type, and normalization using the RMA16 summarization algorithm. Differentially expressed genes were determined after filtering the data sets for background level of fluorescence (as determined by the Expression Console). Differentially expressed transcripts were determined by comparing normalized average intensities (log2 GC-RMA signal intensity values) of biological triplicates of MCLR-treated larvae to control sample groups and filtering for statistically significant genes using an unpaired Student’s *t*-test and the cut-off value of *p* < 0.05, and fold change of 1.5. Microarray data have been deposited to the Gene Expression Omnibus (GEO) database under the accession number (GSE73739).

### 4.6. Quantitative Real-Time PCR 

Quantitative real time PCR was performed on a Lightcycler capillary system 2.0 (Roche, Indianapolis, IN, USA) with SYBRgreen fluorescent label (PrimerDesign, Chandler’s Ford, UK). cDNA samples were synthesized as described in Section 4.4. The 10 μL qPCR reaction mix contained 5 μL of 2× Precision SYBRgreen mastermix (PrimerDesign), 0.25 μL of 10 μM of each PCR primer, 2 μL of diluted cDNA template (12-fold), and 2.5 μL of RNase-free water. The qPCR parameters consisted of initial denaturation at 94 °C for 15 min followed by 45 cycles at 94 °C for 15 s, 60 °C for 20 s, and 72 °C for 10 s. All reactions were performed in triplicates. To ensure that only one PCR product was amplified and that stock solutions were not contaminated, a non-template control was included and melting curve analysis was performed at the end of the amplification phase. Standard curves were constructed for each gene using serial dilutions of stock cDNA. The primers of the target genes were designed based on *Danio rerio* gene sequences found in the NCBI. Gene names, accession numbers, and forward and reverse primer sequences are shown in Table 2. Gene expression levels were quantified relative to the expression of reference genes *gap43* and *rpl13* (geNorm reference kit, PrimerDesign) using the qbase PLUS analysis program (Biogazelle, Gent, Belgium). This software uses a modified classic ΔΔ*C*_t_ (cycle threshold) method to take multiple reference genes and gene specific amplification efficiencies into account.

For the statistics of qPCR analysis, we have used the one-way ANOVA *F*-test (when equality of variances is assumed) or the Welch test (when unequal variances is assumed), followed by an appropriate post-hoc test (Tukey test in the case of equal variances and Dunnett’s test for the case of unequal variances). The hypothesis of equality of variances was checked by Levene’s test for equality of variances.

### 4.7. Behavior Assay

Three dpf larvae were exposed to 100 μg/L and 500 μg/L of MCLR, as well as to 0.05% ethanol as control, for four days in 250 mL-glass beakers. At seven dpf, larvae were placed in a 96-well plate, one larva per well, 30 larvae per group. The plate was introduced in the DanioVision automated tracking system powered by EthoVision (Noldus, Wageningen, The Netherlands). This system allows tracking locomotor activity of larvae under controlled temperature and lighting conditions. Temperature was set at 28.5 °C and the lighting was variable during tracking period to analyze larvae response to external stimuli. After applying 10 min dark and 10 min light conditions in order to let larvae habituate to the system, tracking was performed at two rounds of 10 min light/10 min dark phases. Total duration of the tracking was 40 min. The mean of the total distance covered by larvae in each group was measured in 2 min time bins. Statistical analysis was done by two-factor mixed-design ANOVA for comparing the mean differences between groups taking into account time (“within-subjects” factor) and MCLR concentration (“between-subjects”) factor.

## Figures and Tables

**Table 1:** Microarray analysis. List of differentially expressed genes top ranked based on fold-change that were differentially expressed, by microarray analysis, upon exposure of three days post-fertilization (dpf) zebrafish larvae to 500 μg/L MCLR for four days, *p* ≤ 0.05.

Transcript ID	Gene Symbol	Gene Description	NCBI Gene ID	*p*-Value	Fold Change
		Upregulated genes			
13122067	*tm6sf2*	transmembrane 6 superfamily member 2	791179	3.21 × 10^−2^	2.95
12945361	*zgc:194355 (lrat-like)*	zgc:194355 (lecithin retinol acyltransferase-like)	556575	3.15 × 10^−2^	2.65
13223317	*rdh20*	retinol dehydrogenase 20	555864	1.42 × 10^−2^	2.42
13085946	*rpe65a*	retinal pigment epithelium-specific protein 65a	393724	7.28 × 10^−3^	2.39
12985711	*gch2*	GTP cyclohydrolase 2	64263	9.80 × 10^−4^	2.14
13263303	*dct*	dopachrome tautomerase	58074	1.50 × 10^−2^	2.13
13156297	*rlbp1b*	retinaldehyde binding protein 1b	402990	6.80 × 10^−3^	2.13
13135621	*pmelb*	premelanosome protein b	562810	6.42 × 10^−3^	2.06
13305424	*slc16a8*	solute carrier family 16 (monocarboxylate transporter), member 8	557884	3.47 × 10^−2^	1.96
13112995	*LOC103911087*	uncharacterized LOC103911087	103911087	7.87 × 10^−3^	1.95
12968503	*inpp5kb*	inositol polyphosphate-5-phosphatase Kb	566188	2.05 × 10^−3^	1.91
13126385	*rdh5*	retinol dehydrogenase 5 (11-*cis*/9-*cis*)	556528	3.12 × 10^−2^	1.91
13279630	*stra6*	stimulated by retinoic acid 6	724007	2.31 × 10^−2^	1.73
12972154	*pmela*	premelanosome protein a	321239	1.63 × 10^−3^	1.72
12946322	*tyrp1b*	tyrosinase-related protein 1b	437022	6.72 × 10^−5^	1.70
13284986/ 13022340	*tyr*	tyrosinase	30207	9.79 × 10^−3^ 5.54 × 10^−3^	1.69 1.55
13156897	*stra6*	stimulated by retinoic acid 6	724007	7.76 × 10^−3^	1.68
12988859	*si:ch1073-13h15.3 (LOC563241)*	si:ch1073-13h15.3 (putative all-trans-retinol 13,14-reductase)	563241	2.07 × 10^−2^	1.68
13109427	*slc45a2*	solute carrier family 45, member 2	558311	3.11 × 10^−2^	1.66
13129757	*LOC101882639*	uncharacterized LOC101882639	101882639	3.57 × 10^−2^	1.66
13133349	*LOC566587*	Erb-b2 receptor tyrosine kinases (ERBB) receptor feedback inhibitor1-like	566587	1.79 × 10^−2^	1.60
13182209	*si:dkey-31f5.1 (slc26a3.2)*	solute carrier family 26 (anion exchanger), member 3, tandem duplicate 2	563896	9.96 × 10^−3^	1.59
13274399	*bco1l*	β-carotene oxygenase 1, like	393580	2.98 × 10^−2^	1.57
12994027	*rgrb*	retinal G protein coupled receptor b	554142	3.02 × 10^−2^	1.56
13054709	*LOC100535423*	urokinase plasminogen activator surface receptor-like	100535423	1.69 × 10^−2^	1.56
13216589	*mct1b (slc16a1a)*	solute carrier family 16 (monocarboxylate transporter), member 1a	100534752	4.29 × 10^−2^	1.55
13233501	*tyrp1a*	tyrosinase-related protein 1a	100333145	7.52 × 10^−4^	1.51
13071881	*slc39a4*	solute carrier family 39 (zinc transporter), member 4	562762	4.36 × 10^−2^	1.51
12958158	*zgc:154142*	zgc:154142	555481	1.50 × 10^−2^	1.50
13215240	*oca2*	oculocutaneous albinism II	567419	5.33 × 10^−3^	1.50
Downregulated genes					
13089117	*sepp1b*	selenoprotein P, plasma, 1b	791479	5.75 × 10^−3^	0.66
13124494/ 13279064	*cp*	ceruloplasmin	84702	7.16 × 10^−3^ 2.04 × 10^−2^	0.66 0.66
12993720	*LOC568930*	uncharacterized LOC568930	568930	3.97 × 10^−2^	0.64
13185986	*LOC100331497*	U2 small nuclear ribonucleoprotein auxiliary factor 35 kDa subunit-related protein 1-like	100331497	1.11 × 10^−3^ 4.63 × 10^−2^	0.64 0.56
13269248/ 13285056	*ttn.2 (ttna)*	titin, tandem duplicate 2 (titin a)	317731	1.54 × 10^−2^ 4.63 × 10^−2^	0.63 0.56
13269083	*ttnb*	titin b	100001684	1.40 × 10^−2^	0.63
13070223	*si:ch211-250g4.3*	si:ch211-250g4.3	557772	3.90 × 10^−2^	0.63
12959767	*LOC100330916*	uncharacterized LOC100330916	100330916	3.61 × 10^−2^	0.60
13283642	*si:dkey-7c18.24*	si:dkey-7c18.24	562950	3.36 × 10^−3^	0.60
13037425	*si:dkey-8k3.2*	si:dkey-8k3.2	794635	3.54 × 10^−2^	0.60
13191393	*LOC100004951*	stonus toxin subunit β-like (neoverrucotoxin subunit beta-like)	100004951	2.96 × 10^−2^	0.60
13002727	*si:ch211-270n8.1*	si:ch211-270n8.1	792467	3.27 × 10^−2^	0.59
13283794	*zgc:172075*	zgc:172075	555875	4.83 × 10^−2^	0.58
13104731	*si:dkey-1j5.4*	si:dkey-1j5.4	563949	3.26 × 10^−3^	0.54
13072283	*si:ch211-133n4.9*	si:ch211-133n4.9	100000061	2.22 × 10^−2^	0.53
12993635	*zmp:0000001031 (LOC568241)*	zmp:0000001031 (uncharacterized LOC568241)	568241	3.33 × 10^−2^	0.44
13282574/ 13284122	*lgals1l1*	lectin, galactoside-binding, soluble, 1 (galectin 1)-like 1	326706	4.84 × 10^−3^ 4.84 × 10^−3^	0.33 0.33
13076755	*grn2*	granulin 2	336575	4.55 × 10^−2^	0.32
13121248	*krt96*	keratin 96	321502	3.79 × 10^−2^	0.26

NCBI: National Center for Biotechnology Information.

**Table 2:** List of primers used for qPCR analysis.

Gene Symbol	Gene ID	Forward Primer (5′–3′)	Reverse Primer (5′–3′)
*stra6*	724007	CAAGCAATTGTTGTGTTTTGTGTC	TGGTGGGATAACTTCGACAGG
*lrata*	553239	TACGCGTCGATTCGGTTGAA	TTACAAAACTTGTCGGTCTGCTC
*rpe65a*	393724	AGAGACGGGACGGTCTACAA	CCGTCATCCCAAAACTGTGC
*rlbp1a*	393678	TTGAACATCTGACTGTGAAAGACC	GCCTGCCTTGTCTTTAATCATGG
*rlbp1b*	402990	TGAGCTTGCTAAAGGTGTTCAGG	TCAGGATAATCCCGTCTGAAGC
*rgrb*	554142	GGAGCTTTAAAACGCGCACA	CTCTCGAACCCTGAGGAACG
*inpp5k*	566188	GGTTTGTATAAGCCATAGCAAGATG	GTGCAATCTGAAGGACTCTCTG
*tyr*	30207	GCGCTGGAAGGTTTTGCTAAT	AAATGGGGTCGTTGGCAGAT
*dct*	58074	TGTCTAAAGAGTGCTGCCCG	CCGGCAAAGTTTCCAAAGCA
*pmela*	321239	CTCCTGCTCCAGTTACAGATGA	CGTTGGCTACAACTCCCTCC
*pmelb*	562810	CACACAGTTTCACGAAGGCG	GCCAGTATTTGCCCCAGGTT
*cp*	84702	GAAAGAAAGCCCAGGCAACG	ATATCGGCGGTCCTCTCCTT
*sepp1b*	791479	TCTACAGTGGTTGAAGTCCAGC	TCCTCGAACCACTGCTTTCC
*tuba1b*	373080	AATCACCAATGCTTGCTTCGAGCC	TTCACGTCTTTGGGTACCACGTCA
